# Major Thalassemia, Screening or Treatment: An Economic Evaluation Study in Iran

**DOI:** 10.34172/ijhpm.2021.04

**Published:** 2021-02-03

**Authors:** Firooz Esmaeilzadeh, Batoul Ahmadi, Sajad Vahedi, Saeed Barzegari, Abdolhalim Rajabi

**Affiliations:** ^1^Department of Public Health, School of Public Health, Maragheh University of Medical Sciences, Maragheh, Iran.; ^2^Department of Health Management & Economics, School of Public Health, Tehran University of Medical Sciences, Tehran, Iran.; ^3^Department of Healthcare Administration, School of Public Health, Ahvaz Jundishapur University of Medical Sciences, Ahvaz, Iran.; ^4^Department of Health Information Technology, Amol Faculty of Paramedical Sciences, Mazandaran University of Medical Sciences, Sari, Iran.; ^5^Department of Health Management and Social Development Research Center, Faculty of Health, Golestan University of Medical Sciences, Gorgan, Iran.; ^6^Department of Environmental Health Research Center, Faculty of Health, Golestan University of Medical Sciences, Gorgan, Iran.

**Keywords:** Beta-Thalassemia, Cost Analysis, Mass Screening, Treatment, Economic Evaluation, Iran

## Abstract

**Background:** Beta-thalassemia minor and thalassemia major are an autosomal recessive disease with hypochromic, microcytic anemia, and morbidities, Today, therapeutic advances have significantly improved the life expectancy of thalassemia major patients, but at the cost of financial toxicity. The present study aimed to investigate the possibility of increasing the funding for thalassemia screening programs and comparing the cost-effectiveness of screening for thalassemia in the treatment of the patients.

**Methods:** In this study, screening for thalassemia minor was compared with the treatment of thalassemia major patients. A decision tree model was used for analysis. A hospital database, supplemented with a review of published literature, was used to derive input parameters for the model. A lifetime study horizon was used and future costs and consequences were discounted at 3%. The approach of purchases of services was used to evaluate the screening test costs for patients with thalassemia major. Also, a bottom-up method was applied to estimate other screening and treatment costs. All the costs were calculated over one year. The number of gained quality-adjusted life years (QALYs) was calculated using the EQ-5D questionnaire in the evaluated patients.

**Results:** In this study, 26.97 births of patients with thalassemia major were prevented by screening techniques. On the other hand, total screening costs for patients with thalassemia major were estimated equal to US$ 879879, while the costs of preventing the birth of each thalassaemia major patient was US$ 32 624 by screening techniques. In comparison, the cost of managing a patient with thalassemia major is about US$ 136 532 per year. The life time QALYs for this is 11.8 QALYs. Results are presented using a societal perspective. Incremental cost per QALY gained with screening as compared with managing thalassaemia major was US$ 11 571.

**Conclusion:** Screening is a long-term value for money intervention that is highly cost effective and its long-term clinical and economic benefits outweigh those of managing thalassaemia major patients.

## Background

**Key Messages**

** Implications for policy makers**

- Screening for minor thalassemia is more cost-effective than treating thalassemia major patients and the net benefit generated by screening rather than treatment can be used to expand the screening program.
- According to the sensitivity analysis, funding for thalassemia minor screening should be based on the variables affecting the cost-effectiveness of screening in different regions, including the prevalence of minor thalassemia among couples, fertility rates, etc. Hence, screening in areas with high rates of thalassemia minor prevalence and fertility (such as Sistan and Baluchistan) is much more cost-effective than in areas with low minor thalassemia prevalence and fertility rates (like Tehran). So, new cases of the disease should be prevented by allocating more funds and expanding screening programs.
- Given that non-marriages of minor thalassemia couples in later generations may decrease the incidence of minor thalassemia, this policy can be excluded from the screening program in cities where couples undergo all complementary tests.

** Implications for the public**

Treating major thalassemia patients is cost-effective, but it is very close to the cost-effectiveness threshold, and although a large proportion of the costs are paid by the government and insurance companies, the costs imposed on the patients are still high and, in many cases, the patients encounter problems with buying services, especially medication. Screening is more cost-effective than treatment, but the cost of supplementary testing for couples is very high, which may cause the screening procedure to fail and increase the incidence of thalassemia. Therefore, not only the government and insurance companies should pay the costs of the complementary tests, necessary training should also be given to at-risk couples in order to understand the importance of the tests.

Thalassemia is a common genetic disease associated with global health problems.^1^ Compared with other regions, Iran is one of the countries with a higher prevalence of thalassemia.^2^ Beta-thalassemia is caused by mutations in the beta-globin gene on chromosome 11, resulting in decreased or non-synthesized beta chains, leading to severe anemia.^3-5^

**Figure 1:**
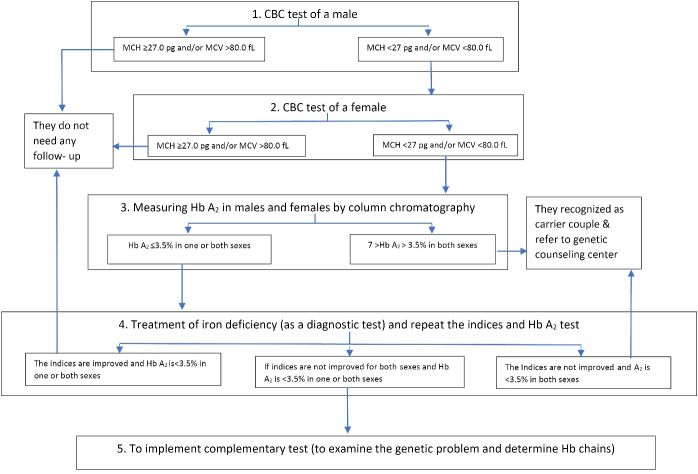
Iranian National Program for Thalassemia Screening.^15^ Abbreviations: CBC, complete blood count; MCV, mean corpuscular volume; MCH, mean corpuscular hemoglobin; Hb, hemoglobin.

 The advances in the treatment of patients with thalassemia major have made it a chronic disease that requires lifelong care.^6-8^ Caring for patients with thalassemia incurs many costs, including laboratory tests, blood transfusions, purchase of iron-chelating drugs, treatment of side effects, regular medical visits, and indirect costs (such as opportunity costs).^9-11^ Despite the costly and painful medical treatments for thalassemia major patients and complications such as appearance changes, endocrine problems, chronic liver diseases, growth disorder, osteoporosis, etc are still threatening various aspects of the lives of the patients and their families and may adversely affect their physical and mental health and quality of life.^12-14^ Screening for thalassemia minor to reduce the incidence of thalassemia major can be considered as one of the most effective ways to reduce the cost of treatment. Minor thalassemia screening programs have been implemented in Iran since 1997. Thus, for the official registration of marriage, the result of a thalassemia screening test is required. Therefore, the cellular blood count (CBC) test was performed on men in the first phase. Then, if a minor problem with thalassemia in men is suspected, a CBC test will be performed on women. On the other hand, if both men and women suspect minor problems with thalassemia, other tests should be performed. Details of this process are provided in Figure 1.^15^

 Considering the high treatment costs and the morbidity of the disease in the limited-resource setting, this study aimed to answer the following question: “How much should be spent on reducing the incidence of thalassemia major through spreading the screening program for thalassemia?”

## Methods

 In this study, we compared the cost-effectiveness of thalassemia screening with the treatment of patients with thalassemia major, so that we considered screening costs as the cost and treatment costs of patients with thalassemia major as the effectiveness of thalassemia screening (Screening costs to prevent the birth of children with thalassemia major). In other words, screening methods prevented the birth of babies with thalassemia major, which led to a reduction in treatment costs. Also, due to the necessity of treatment patients with thalassemia major, we also estimated the cost-utility of treating patients in which the cost of treating patients as a cost and the quality-adjusted life year (QALY) created by treatment as the effectiveness of treating patients with thalassemia was considered.

###  Screening Costs

 In this study, in 2015, all officially married couples (67 089 couples) in Tehran were screened for thalassemia (Figure 1). Thus, mean corpuscular volume (MCV) and mean corpuscular hemoglobin (MCH) tests for men were measured in the first step. In these tests, MCV ≥80 and MCH ≥27 indicate that couples can get married. If the above results were not valid, MCV and MCH were measured for women. Other additional tests, if necessary, are shown in Figure 1. The cost of direct screening for couples was measured to estimate the cost of screening based on the type of test performed by each person as a non-tariff measure. Indirect costs include travel (transportation) costs and missed opportunity costs. The couples who participated in the study evaluated these costs by filling out a checklist. In addition, the mean cost of thalassemia tests on fetuses suspected of thalassemia major and abortions was estimated by examining the cost of tests on all suspected fetuses and miscarriages due to thalassemia major and indirect costs during 2014 and 2015.

###  Screening Effectiveness

**Figure 2:**
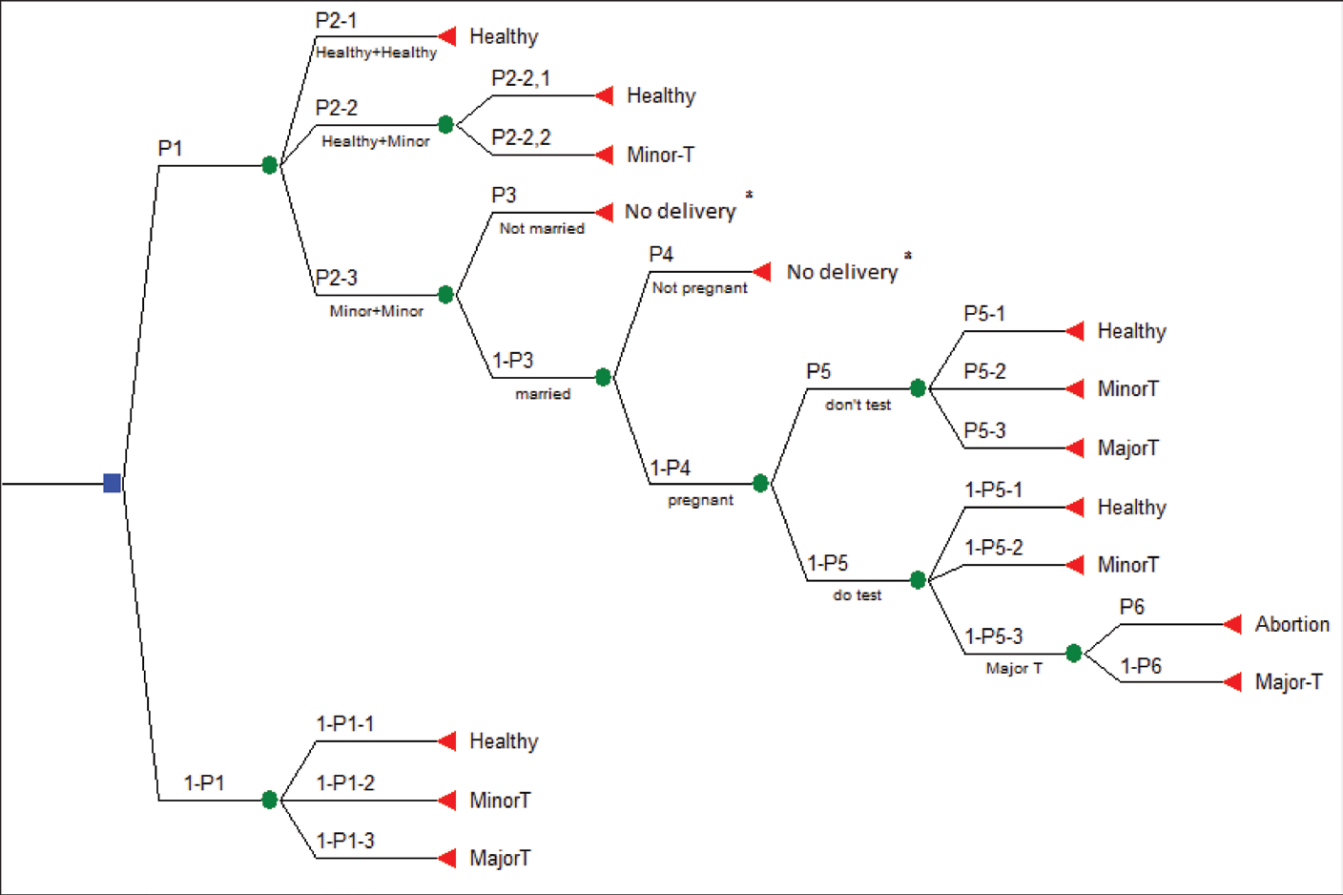
Decision Tree Model. N= *n*_1_ + (TFR-1)* *n*_2_ n_1_ = [(N_COS_ * P_BCM_ * P_MTM_ * P_MCHCh_ * P_PND2 _* P_MT_ * P_OB_) + (N_COS_ * P_BCM_ * P_NMTM_ * P_MT_)]
*n*_2 _=[((*n*_1_ - (*N*_COS_ * *P*_BCM_))* *P*_MTM_ *P_MCHCh_*P_PND2 _* P_MT_ * P_OB_)+ ((*n*_1 _– (*N*_COS_ * *P*_BCM_)* * P_NMTM_ * P_MT_)] N = the number of children with thalassemia major prevented by screening; *n*_1_ = the number of children with thalassemia major prevented by screening in the first pregnancy; *n*_2_= the number of children with thalassemia major prevented by screening in the second pregnancy; TFR = total fertility rate; *P*_MTC_= probability that the couple are both thalassemia minor; *N*_COS_ = number of couples referring for thalassemia screening; *P*_BCM_ = probability of the both couples being minor; *P*_MTM_ = probability that thalassemia minor men and women marry while they are aware of their problem; *P*_MCHCh_ = probability of the both couples being minor; *P*_PND2_ = probability of doing PND2 tests on an embryos of married minors; *P*_MT_= probability of the birth of a thalassemia major child from a minor couple; *P*_OB_= probability of abortion of thalassemia major embryos; *P*_NMTM_ = probability of separation after getting aware of having thalassemia minor; * No delivery: It is a condition in which pregnancy is potentially impossible.

 In order to evaluate the effectiveness of screening, we first used a decision tree model (Figure 2) and estimated the probability of a group of children who were prevented from giving birth to thalassemia through screening. Next, we assumed that if patients with thalassemia major were born, their treatment costs would be the same as those of existing patients. Therefore, we included a sample of existing patients in the study and estimated the cost of their treatment. Finally, the cost of treatment of these patients was considered the effectiveness of screening. Considering the sensitivity of simultaneously performing tests of MCV and MCH equal to 98.5% and the prevalence of thalassemia minor in Tehran equal to 1.9% have been estimated, the probability of a false negative result for thalassemia minor is equal to 0.000285. If the mentioned person marries a person with thalassemia minor, with a probability of 0.019, the couple’s infant will have a 0.25% risk of developing thalassemia major. Therefore, the probability of giving birth to an infant with thalassemia major due to a false-negative result will be equal to 0.0000013. Therefore, the effects of these samples were ignored in the present study. It should be noted that the confirmation of thalassemia minor depends on the repetition of a series of tests performed by the couple (male and female). Therefore, the effects of false positives, which were almost zero, were also ignored in this study.^16,17^

###  Treatment Costs

 In this study, the number of samples required to estimate costs, based on Altman’s nomogram for calculating sample size in the graphical method (28) and the number of independent variables, was calculated at least 170 people.^18^ Therefore, 200 patients were randomly selected and 198 patients included in the study with sufficient knowledge and consent. The bottom-up method and the prevalence-based approach were used to estimate costs. Direct treatment costs include iron chelation therapy, blood transfusions, doctor visits, medications, laboratory tests, and hospitalization costs. These costs were determined through interviews with patients, their family members, and physicians’ tariffs, checking the patients’ records and patients’ health insurance records. Then, the above-mentioned expenses are calculated as the cost of purchasing the service after increasing the insurance premium and the Ministry of Health subsidy. Indirect costs are also estimated based on the determined average spelling time, the time the family spends on face-to-face patient care, or the cost of family caregivers. Furthermore, time spent meeting with healthcare providers, sponsorships, supports, adapting patients to illness conditions via changing decoration, traffic, and other costs were calculated prospectively and with patient follow-up (details of cost estimation are presented in Esmaeilzadeh and colleagues’ study^11^).

###  Treatment Utility

 To estimate the treatment utility of thalassemia patients in this study, the EQ-5D questionnaire weighed by Goodarzi et al^19^ according to Iran’s preferences was used. Therefore, using the EQ-5D questionnaire and the estimated weights in Goodarzi and colleagues’ study, we estimated the QALYs resulted from the patients’ treatment. Given that if the patients did not receive medical care, they would experience very low-utility early years of life and would die early.^12,20^ In this study, the utility without treatment was considered zero and all estimated QALYs were attributed to treatment.

###  Cost–Utility Analysis 

**Figure 3:**
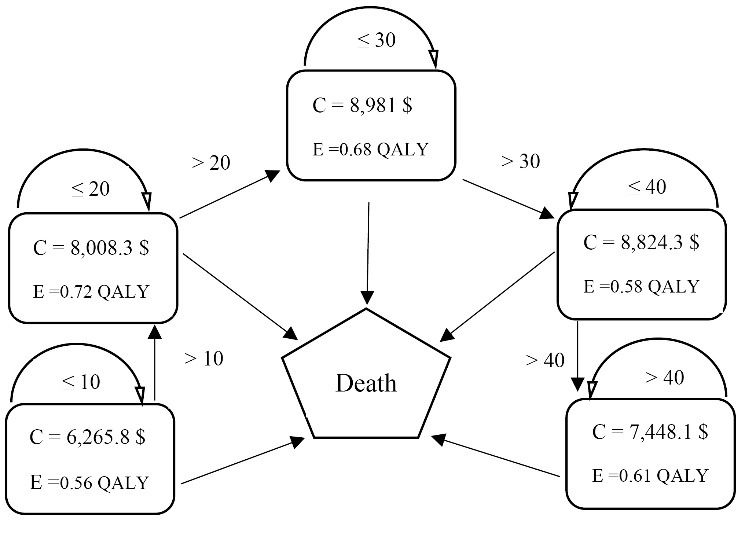
Schematics of the Markov Model. Note: For simplicity, the model is depicted as 10-year age groups. Abbreviation: QALY, quality-adjusted life year.

 In this study, the Markov model was used to estimate the cost- utility of treatment of thalassemia patients. For this purpose, the costs and QALYs of the patient’s treatment were entered into the model annually (Figure 3).

 Accordingly, costs and QALYs gained from patients’ treatment samples were estimated for all studied age groups over a one-year period. In the next step, to estimate the costs and QALYs of patients’ treatment throughout their lifetime, all patients entered in the Markov model by age groups of zero to one. In the following, patients who were alive at the end of the first year were identified (Table 1). Finally, the cost of the first year and the number of QALYs are estimated based on the cost- utility of the zero to one age group (Table 2). Similarly, the cost and QALY value for the next few years are estimated (until all patients die and are excluded from the study model), then they are added to the first year cost and QALY is obtained at a discount rate of 3%.

**Table 1:** Decision Tree Probabilities

Number of couples screened in 2014 in Tehran (couples)^a^	67 089
Probability of the both couples being minor (*P*_BCM_ = *P*_2_3_)^a^	0.00
Probability of marrying minor couples (*P*_MTM_* = *1 *- P3*)^a^	0.90
Probability of minor couples want to have children (*P*_MCHCh_* = *1 *– P*4)^a^	1.00
Probability of doing PND2 tests on an embryos of married minors (*P*_PND2_* = *1 *– P*5)	1.00
Probability of the birth of a thalassemia major baby from a minor couple (*P*_MT_ =1 *– P*_5_3_)	0.25
Probability of abortion of thalassemia major embryos (*P*_OB _=P6)^a^	1.00
Total fertility rate in Tehran^b^	1.41

Abbreviation: PND2, Frequency of genetic testing 2. ^a^Extracted from study samples; ^b^Source: Statistical Centre of Iran.

**Table 2:** Probability of Annual Thalassemia Patient Death Rate in Different Age Groups^28^

**Age Groups**	**Probability of Annual Death**
0-1	0.012
2-5	0.003
6-10	0.002
11-15	0.016
16-20	0.025
21-30	0.015
>30	0.345

###  Sensitivity Analysis

 Sensitivity analysis was performed on the variables that affect the results of the study within an acceptable range (Table 3). Also, couples’ marriage with thalassemia minor could affect the results in the present study if PND (frequency of genetic

 testing) tests were not performed on the fetuses. Accordingly, the above feasibilities were also considered in the present study. According to Table 4, the cost-effectiveness of the treatment result is estimated with an acceptable discount rate.

**Table 3:** Direct and Indirect Costs of Screening for Thalassemia in Tehran (N = 134 178)

**Cost Sources**	**Fees for One Year of Screening (Dollar)**			**Cost Share Of Total Costs (%)**
	**Costs of Doctors’ Visits**	**Costs of Tests**	**Total**	
Male CBC	208 932	68 130	277 062	31.5
Female CBC	23 438	7643	31 081	3.5
Hb A2	5581	6247	11 827	1.3
Costs of supplementary tests		30 102		3.4
PND1 cost		143 917		16.4
PND2 cost		43 556		5.0
Abortion costs		8039		0.9
Costs of visiting medical centers		37 776		4.3
Costs of lost time		296 518		33.7
Total costs		879 879		100
Number of thalassemia majors prevented by screening 67 089 couples				26.9

Abbreviations: CBC, cellular blood count; Hb A2, hemoglobin A2; PND1, Frequency of genetic testing 1; PND2, Frequency of genetic testing 2.

**Table 4:** Sensitivity Analysis of Variables Affecting Cost-Effectiveness of Screening for Thalassemia

**Intended Variable for Sensitivity Analysis**	**Variable Value for Sensitivity Analysis**	**Screening Cost**	**Number of Patients Prevented by Screening**	**Costs Per Prevented Patient**
Base mode^a^	0.00	879 879	26.97	32 624
Probability of the couple to be minor both	0.00	865 995	19.71	43 937
	0.00	895 333	35.06	25 537
	0.00	979 130	78.84	12 419
	0.00	1 115 802	140.14	7962
	0.01	1 277 565	219.08	5831
General fertility rate	1.22	873 925	23.86	36 627
	1.81	891 785	33.19	26 869
	2.00	897 739	36.31	27 048
	3.00	920 048	47.92	25 339
	4.00	936 832	56.75	19 550
Probability of minor couples not to marry	0.05	882 298	26.97	32 714
	0.31	870 199	26.97	32 265
	0.52	860 520	26.97	31 907
Probability of undergoing PND2 tests	0.92	874 849	24.69	35 433
	0.81	869 809	22.41	38 813
	0.71	864 758	20.12	42 980
Probability of minor couples not to marry	0.10	879 879	26.97	32 624
Probability of undergoing PND2 tests	1.00			
Probability of minor couples not to marry	0.11	859 696	17.74	48 461
Probability of undergoing PND2 tests	0.62			
Probability of minor couples not to marry	0.52	859 696	17.74	48 461
Probability of undergoing PND2 tests	0.61			

Abbreviation: PND2, Frequency of genetic testing 2. ^a^ In the base mode, the probability of the couple to be both minor was 0.0012, the probability of the couples not to marry was 0.1, the probability of undergoing PND2 test was 1, and the general fertility rate was 1.4.

## Results

###  Screening Cost

 All the couples referring for premarital tests in Tehran were included in the present study (67 089 couples = 134 178 individuals). One percent and 3.2% of the male and female participants were under 18 years old, 90.3% and 90.9% were 18-35 years old, and 9.6% and 5.9% were over the age of 35, respectively. In addition, 0.2% of men and 0.1% of women are illiterate, 37.3% and 23.2% have basic education to diploma, and 76.6% and 62.6% had university degrees, respectively.

 As shown in Table 3, the cost of screening for thalassemia minor for 67 082 couples was US$879 879, most of which was the time spent by couples in counseling service providers. Amongst the services received by the couples, men’s CBC and abortions were the most and the least costly ones, respectively. In addition, 27% of the cost of screening is related to the visiting doctor. According to the decision tree model and the probability obtained from the sample, screening 67 089 couples can prevent the birth of 26.97 major thalassemia patients. Therefore, the cost of screening to prevent delivery of each major patient with thalassemia is US$32 624.

 According to Table 4, an increase in the probability of minor couples and the general fertility rate would lead to increased costs, and a greater number of prevented patients with screening. However, the percentage increase in patients identified for prevention is much higher than the cost of screening. Therefore, the cost of screening for each patient will be greatly reduced. A decreased probability of undergoing PND2 tests would also lead to an increase in the number of diagnosed patients and the cost of identifying each patient as well.

 Increased probability of thalassemia minor when all couples are tested for PND2, their withdrawal from marriage has no significant impact on the cost of disease prevention, however, when the possibility of PND2 testing decreases, the impact of withdrawal from marriage increases, which will significantly reduce the cost of birth for each thalassemia major patient.

###  Treatment Costs for Patients With Thalassemia Major (Screening Effect)

 In this study, the mean ages of male and female thalassemia major patients were 23.3 ± 9.7 and 25.8 ± 9.9 years, respectively, and 51.5% of the participants were men. The patients had received an average of 30.19 bags of blood, 725.2 vials of deferoxamine, 404.9 deferasirox, and 717.8 deferiprone a year.

 According to Table 5, treatment of each patient with thalassemia major cost an average of US$8321.8 per year, most of which was associated with the drugs used by the patients, so that they accounted for over 60% of the costs. In the next place came injections and blood transfusions. In contrast, splenectomy is the lowest cost, accounting for 0.54% of the total cost.

**Table 5:** Mean Annual Costs of Treatment for Any Thalassemia Major Patient by Cost Sources

**Type of Costs**	**Average Annual Cost (%)**
Blood	1118.0 (13.43)
Medical visits	182.9 (2.20)
Nursing services	303.7 (3.65)
Laboratory services	136.6 (1.64)
Diagnostic services	216.0 (2.61)
Medicine	5026.4 (60.40)
Deferoxamine pump and other consumer items in home	155.0 (1.86)
Hospitalization	103.4 (1.24)
Splenectomy	44.8 (0.54)
Going to the service providing centers	205.3 (2.47)
Transportation	69.8 (0.84)
Lost opportunities for patients	356.2 (4.28)^a^
Lost opportunities for patients’ families	217.4 (2.61)
Building rent and other costs related to buildings	186.3 (2.24)
Lost welfare	^b^
Total	8321.8 (100.00)

^a^ In 2015, samples of patients under 15 years had lost 810 working days to get the services. Given that this age group was not included in the working age, changing it to monetary costs was refused. ^b^ According to Naghavi et al,^29^ years lost due to disability associated with thalassemia is 25%. Given the per capita gross domestic product of US$5442 for Iran^30^ in 2014 it can be said that, in addition to the costs expressed in the table, almost all patients lose US$ 1360.5 a year for costs associated with pain and suffering caused by the disease.

 According to Table 6, the cost of treatment for patients with thalassemia major in the 21-31 age group is higher than that of other age groups. In contrast, the QALYs from treatment were greater in the age group 11-20 year-old compared to other age groups. In addition, for patients in the 11-20 and 31-40 age groups, the cost of each QALY is the lowest and the highest, respectively.

**Table 6:** Mean Annual Cost and QALYs Produced by Treatment of Any Thalassemia Patient by Age Group

**Age Groups**	**Average Annual Cost**	**Average Annual QALY**	**Cost Per QALY**
0-10	6265	0.56	11 188
11-20	8008	0.72	11 122
21-30	8981	0.68	13 207
31-40	8824	0.58	15 213
>40	7448	0.61	12 209

Abbreviation: QALY, quality-adjusted life year.

 According to Table 7, the mean cost of any patient’s lifetime at a discount rate of 3%, regardless of the costs of welfare lost and opportunity lost due to early death was US$136 532, which generated 11.8 QALYs during their lifetime. Thus, the cost of creating each QALY was US$11 571. The results of the sensitivity analysis (Table 7) showed that an increase in the discount rate would decrease the cost per QALY and increase the effectiveness of the program.

**Table 7:** Mean Cost and QALYs Produced With Treatment in the Lifetime of Any Thalassemia Major Patient

**Discount Rate**	**Average Lifetime Cost**	**Average Lifetime QALY**	**Cost Per QALY**
0%	209 071	17.8	11 746
3%	136 532	11.8	11 571
5%	107 415	9.4	11 427
6%	96 415	8.5	11 343

Abbreviation: QALY, quality-adjusted life year.

## Discussion

 Having applied a discount rate of 3% in this study, we estimated the cost of preventing the birth of any major thalassemia patient through screening to be US$32 624. In addition, the lifetime treatment cost of any thalassemia major patient was US$136 532, regardless of the loss of welfare and opportunity due to early death, resulting in 11.8 QALYs in the lifetime. Therefore, the cost of generating each QALY would be US$11 571. Considering three times the per capita gross domestic product (US$16 325),^21^ as the effectiveness threshold of each QALY, it can be considered that the treatment of patients with severe thalassemia is cost-effective. According to the results of this study, the cost of treatment per patient is more than four times the cost of preventing each patient from giving birth; so, even without considering the costs of welfare lost and opportunity lost due to the early death, screening was much more cost-effective than the treatment of the patients, and generated a net profit of US$103.908 per preventing the birth of any patient.

1. As in the present study, several studies^10,22-24^ showed that screening was more effective than patient treatment. The cost of preventing the birth of a thalassemia major patient and the annual cost of treatment for each patient in the present study and the aforementioned ones were different, the reasons for which could be the difference in the prevalence of the disease, the cost of lost opportunity, tariffs for services, the dosage and type of the drugs used, and the people’s beliefs. Some studies carried out in Iran,^25^ Taiwan^26^ and Thailand^27^ estimated the annual treatment costs for thalassemia patients were US$2252.7, US$7464.4, and US$950, respectively. The lower treatment costs in studies conducted in Iran and Thailand may be due to the low utilization of services, especially iron-chelating drugs. In other words, in the above studies, the mean number of drugs used by each patient was much lower than the drugs in this study. A sensitivity analysis of the variables that influence the cost-effectiveness of thalassemia screening (Table 4) highlights the following important aspects for policy-makers: An increase in the probability of minor couples (prevalence of thalassemia minor in the community), with the assumption of stability in other parameters, will increase the number of thalassemia major patients prevented with screening, and the costs per prevented patient will be significantly reduced. Therefore, in regions and countries with high prevalence of thalassemia minor and high incidence of thalassemia major, governments can increase the screening rate up to several times as much in order to have better coverage of the program.
2. In areas where nearly 100% of the couples undergo PND2 test and abortion of thalassemia major embryos, increased probability of withdrawal of minor couples from marriage may not significantly affect the outcome of cost-effectiveness, and given the fact that it can create a psychological burden in the community and increase the number of minors in future generations, it seems that screening policies can be eliminated, but in areas where couples do not undergo PND2 tests and abortion of thalassemia major embryos, this can be very effective in the prevention of thalassemia major and make the program more effective as well.
3. Probability of undergoing PND2 tests and abortions is a parameter that greatly affects the outcomes, so that a decline in each can reduce the effectiveness of screening and increase the cost for effectiveness. In some cases, couples are reluctant to undergo testing and abortion due to the high cost of testing or lack of understanding of the importance of the problem. These problems can be solved by increasing the couples’ information and the insurance coverage of the tests.

The use of transfer payments can also be very helpful in reducing the costs of supplementary tests, as in the present study, 27% of the screening costs were associated with the costs of medical visits, while no visits or counseling was done by doctors for over 90% of the couples (medical counselling was only provided to the couples who were both suspected of thalassemia). On the other hand, the total cost of PND1, PND2 and abortion is about 22% of the cost, but since this is the cost paid by a small number of couples, the cost per couple is very high. In many cases, this may be one of the reasons for the failure of the screening procedure. Given that no service is provided for the money received for doctors’ visits, the money could be considered as transitional payments for supplementary and genetic tests, and the tests can be done for free or at very low costs.

## Conclusion

 In this study, it was found that thalassemia screening is much more effective than treating thalassemia patients and generates net income. Therefore, the net income of each preventive thalassemia major case (estimated in this study) can be used to promote thalassemia screening to reduce the incidence of the disease. In addition, according to the results of the sensitivity analysis, in areas with a higher prevalence of thalassemia minor, the net income from screening has increased significantly compared with treatment. Thus, given the high probability of the incidence of thalassemia major in such areas, more money should be spent on screening for thalassemia than in other areas.

## Ethical issues

 The Tehran University of Medical Sciences ethics committee that has approved the research.

## Competing interests

 Authors declare that they have no competing interests.

## Authors’ contributions

 Conceived and designed the study: FE, BA. Analyzed the data: AR, FE, and SB. Wrote the paper: FE, AR, SV, SB, and BA. All authors have read, and confirm that they meet ICMJE criteria for authorship.
